# Effect of Virtual Reality-Assisted Discharge Education on Readiness for Hospital Discharge Following Transurethral Resection of the Prostate for Benign Prostatic Hyperplasia: A Randomized Controlled Trial

**DOI:** 10.3390/healthcare14172884

**Published:** 2026-09-07

**Authors:** Büşra Şahin, Hilal Hatice Ülkü, Gökhan Şahin

**Affiliations:** 1 Surgical Nursing Department, Faculty of Nursing, Aydin Adnan Menderes University, Aydin 09100, Türkiye; 2 Aydın Vocational School, Aydin Adnan Menderes University, Aydin 09100, Türkiye; hilal.gulludere@adu.edu.tr; 3 Urology Clinic, Aydin State Hospital, Aydin 09100, Türkiye; gokhansahinn7510@gmail.com

**Keywords:** virtual reality, transurethral resection of the prostate (TURP), patient discharge, patient education as topic, perioperative care, randomized controlled trial

## Abstract

**Highlights:**

**What are the main findings?**

- VR-assisted discharge education significantly improved readiness for hospital discharge in patients undergoing transurethral resection of the prostate (TURP).
- Patients receiving VR education showed greater gains in discharge readiness than those receiving routine discharge education.
- A structured, immersive educational intervention can enhance patients’ preparedness for safe postoperative self-management.

**What are the implications of the main findings?**

- VR-assisted education can be incorporated into routine perioperative nursing care to complement conventional discharge education.
- Technology-supported, patient-centered discharge education may improve the quality and consistency of perioperative patient education. These findings support further multicenter trials to evaluate the long-term clinical impact of VR-assisted discharge education.

**Abstract:**

**Background:** Effective discharge education is essential for promoting postoperative recovery following transurethral resection of the prostate (TURP); however, conventional discharge education may be insufficient to adequately prepare patients for self-care after discharge. Virtual reality (VR)-assisted education has emerged as an innovative approach that provides immersive and interactive learning experiences. This study aimed to evaluate the effect of VR-assisted discharge education on readiness for hospital discharge in patients undergoing TURP. **Methods:** A randomized controlled trial was conducted with 68 patients undergoing TURP at a university hospital. Participants were randomly assigned to either an intervention group (*n* = 34), which received standardized VR-assisted discharge education before surgery in addition to routine care, or a control group (*n* = 34), which received routine discharge education alone. Readiness for hospital discharge was assessed using the Readiness for Hospital Discharge Scale–Short Form (RHDS-SF) before the intervention and immediately prior to discharge. Data were analyzed using a two-way mixed-design analysis of variance. **Results:** Mean baseline scores were comparable between groups. Readiness for hospital discharge increased significantly over time in both groups; however, the improvement was significantly greater in the intervention group. Mixed-design ANOVA demonstrated a significant main effect of time (*p* < 0.001) and a significant time × group interaction (*p* < 0.001), indicating that VR-assisted discharge education produced greater improvements in discharge readiness than routine discharge education. The RHDS-SF demonstrated acceptable to good internal consistency across the two measurement points (Cronbach’s α = 0.705 at pre-test and 0.807 at post-test). **Conclusions:** The structured VR-assisted discharge education intervention resulted in greater improvements in perceived readiness for hospital discharge than routine discharge education among patients undergoing TURP. Structured VR-assisted education may be a useful complementary approach to routine discharge education for supporting patients’ preparedness for postoperative self-care. Further multicenter studies with longer follow-up are needed to determine whether these improvements result in sustained clinical benefits.

## 1. Introduction

Benign prostatic hyperplasia (BPH) is one of the most common urological conditions affecting aging men and represents a major cause of lower urinary tract symptoms that negatively affect quality of life. When conservative or medical treatment fails to provide adequate symptom relief, transurethral resection of the prostate (TURP) remains the gold-standard surgical treatment for patients with benign prostatic obstruction [1,2,3]. Despite substantial advances in surgical techniques and perioperative care, successful postoperative recovery depends not only on the technical success of the procedure but also on patients’ ability to effectively manage self-care after discharge, adhere to postoperative recommendations, and safely continue their recovery at home. Therefore, optimizing patients’ preparedness for the transition from hospital to home has become an essential component of high-quality perioperative care.

Following TURP, patients commonly require education regarding urinary catheter management, postoperative bleeding, pain management, adequate fluid intake, medication use, activity recommendations, and recognition of warning signs that require medical attention after discharge [1,2,3,4]. Although TURP is considered a safe and effective procedure, postoperative recovery continues after hospital discharge and requires patients to actively participate in their own care. Inadequate knowledge of postoperative self-management may delay recovery, increase anxiety, contribute to preventable complications, and lead to unnecessary healthcare utilization [2,3]. Successful postoperative recovery therefore depends not only on surgical treatment but also on patients’ ability to appropriately manage catheter care, maintain adequate hydration, adhere to postoperative recommendations, and promptly recognize potential complications [2,3]. Therefore, supporting patients in acquiring the knowledge and skills required for effective self-care has become a fundamental component of postoperative care following TURP [2,5].

Hospital discharge is no longer regarded simply as the end of inpatient treatment but as a critical transition during which patients assume primary responsibility for managing their recovery at home. A safe and successful transition requires patients to be adequately prepared not only from a clinical perspective but also in terms of their physical, psychological, and educational readiness to perform self-care after discharge [6]. Readiness for hospital discharge refers to a patient’s perceived ability to manage self-care, adhere to treatment recommendations, recognize potential complications, and seek timely medical assistance when necessary [7]. Previous studies have shown that higher discharge readiness is associated with better post-discharge outcomes, including improved treatment adherence, fewer readmissions, and greater patient satisfaction [8,9,10]. Therefore, readiness for hospital discharge has become an important indicator for evaluating the effectiveness of discharge education and the quality of transitional care.

Effective discharge education plays a pivotal role in preparing patients for safe self-management after surgery by improving their knowledge, self-care skills, and adherence to postoperative recommendations [4,11]. However, discharge education in routine clinical practice is often limited to brief verbal explanations and written information provided shortly before discharge. This approach may be insufficient for patients undergoing TURP, who are expected to acquire and retain essential information regarding urinary catheter management, postoperative bleeding, symptom monitoring, medication use, adequate fluid intake, activity recommendations, and warning signs requiring medical attention within a limited period of time [12,13]. Furthermore, patient-related factors such as older age, lower educational attainment, and limited health literacy may further reduce patients’ ability to understand, remember, and apply the information provided after discharge [14]. These limitations highlight the need for more engaging, individualized, and patient-centered educational strategies that can improve learning and better prepare patients for recovery at home.

Advances in digital health technologies have created new opportunities to enhance patient education beyond conventional face-to-face teaching. Mobile applications, web-based educational platforms, video-assisted learning materials, and telehealth interventions enable patients to access health information at their own pace, reinforce learning, and actively participate in postoperative self-management [15,16]. Among these approaches, virtual reality (VR) has emerged as an innovative educational tool that provides immersive, interactive, and experiential learning environments. Unlike traditional educational methods, VR allows patients to visualize clinical procedures and self-care practices in a realistic context, thereby improving attention, knowledge retention, engagement, and learning motivation [17,18]. Recent systematic reviews have also demonstrated that VR-based patient education can improve knowledge acquisition, patient satisfaction, learning experiences, and emotional outcomes, including reductions in anxiety, across a variety of healthcare settings [17,18].

Previous studies have demonstrated the benefits of structured discharge education, telephone follow-up, video-assisted education, and digital health interventions in improving patients’ knowledge, self-care behaviors, and postoperative recovery following urological surgery [4,11,12,19]. Likewise, studies conducted in different clinical settings have reported that virtual reality-based educational interventions can enhance patient engagement, knowledge acquisition, and learning experiences [17,18]. However, evidence regarding the effectiveness of virtual reality-based discharge education specifically for patients undergoing TURP remains scarce. Moreover, few randomized controlled trials have examined its impact on patients’ readiness for hospital discharge, which is considered a key indicator of successful transitional care and effective discharge education. Accordingly, the present study aimed to evaluate the effect of a virtual reality-based discharge education program on readiness for hospital discharge in patients undergoing TURP for benign prostatic hyperplasia.

## 2. Materials and Method

### 2.1. Study Design

This study was designed and conducted as a single-center, parallel-group randomized controlled trial involving patients undergoing TURP at a tertiary university hospital in western Türkiye between May and November 2024. The trial was retrospectively registered at ClinicalTrials.gov (NCT07507123) on 30 March 2026. The trial was retrospectively registered because of an administrative oversight regarding prospective trial registration at the time the study was initiated.

### 2.2. Sample Size and Participants

**Figure 1:**
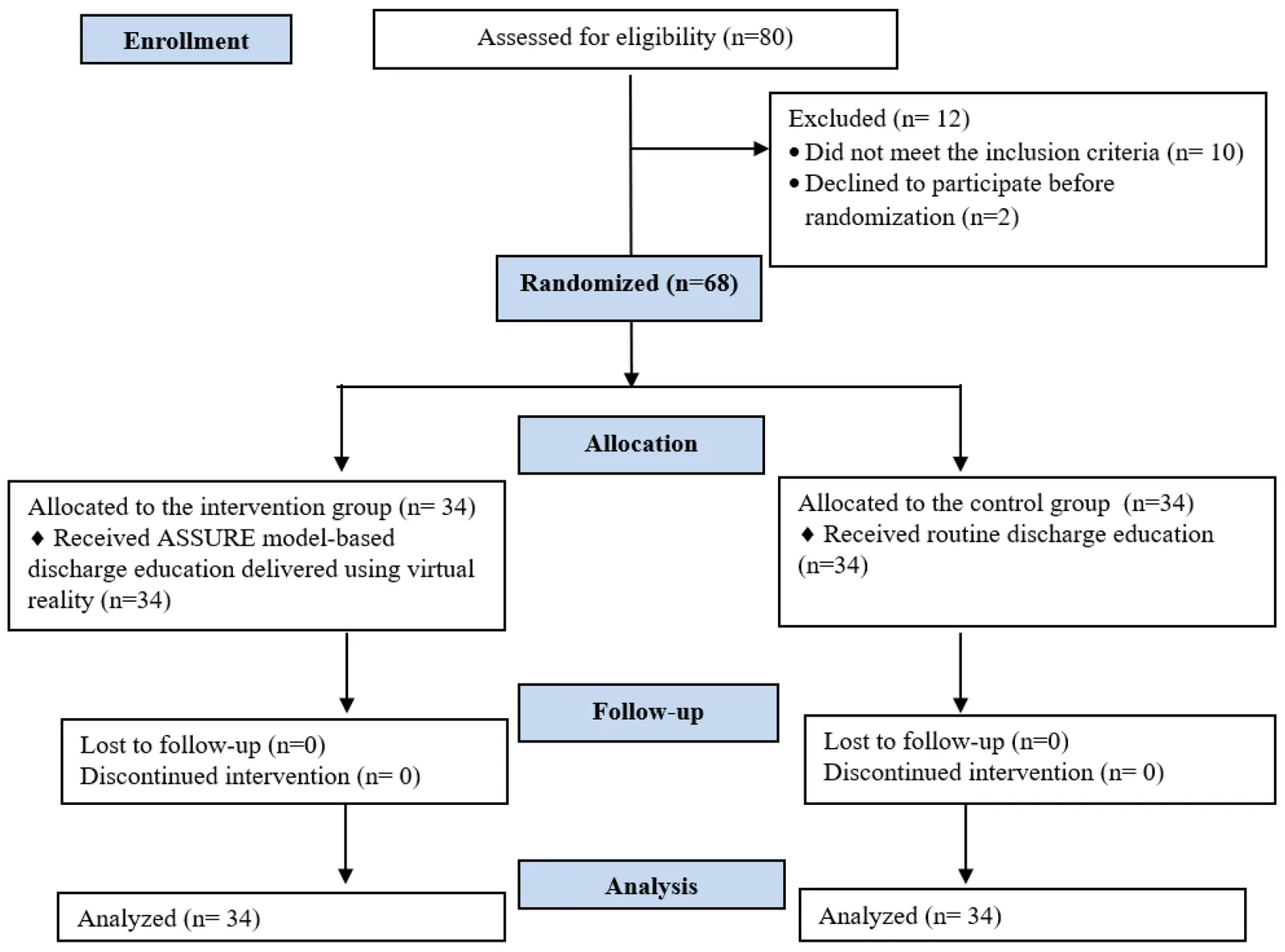
CONSORT flow diagram of participant recruitment, randomization, follow-up, and analysis.

Based on an expected effect size (Cohen’s d = 0.80) reported in a previous randomized controlled trial evaluating a VR-based educational intervention [20], an alpha level of 0.05, a statistical power of 0.80, and a 1:1 allocation ratio between the intervention and control groups, the minimum required sample size was determined as 42 participants, with 21 participants required in each group. Although the previous trial evaluated preoperative anxiety rather than readiness for hospital discharge, it was selected because no published randomized controlled trial reporting an effect size for VR-assisted discharge education in patients undergoing TURP was available at the time the present study was designed. To account for a potential 10% attrition during the study, the target sample size was increased to 46 participants, with at least 23 participants planned for each group. Because recruitment continued throughout the prespecified recruitment period and additional eligible patients became available, 68 participants were ultimately enrolled, thereby exceeding the minimum required sample size while preserving the planned allocation ratio. The inclusion criteria were being over 18 years of age, being literate, and being able to speak and understand Turkish. Patients with visual or hearing impairment, a history of previous TURP, or employment as healthcare professionals were excluded from the study. During the recruitment period, 80 patients were assessed for eligibility. Of these, 12 were excluded before randomization: 10 did not meet the predefined eligibility criteria and two declined to participate before randomization. The remaining 68 eligible participants were randomized in a 1:1 ratio to the intervention (*n* = 34) and control (*n* = 34) groups. All randomized participants completed the study and were included in the final analysis, with no loss to follow-up. The participant recruitment, allocation, follow-up, and analysis process is presented in the CONSORT flow diagram (Figure 1).

### 2.3. Randomization and Allocation

Patients who met the inclusion criteria and agreed to participate were assigned to the experimental and control groups using a computer-generated randomization method. A total of 68 patients were included in the study, with 34 assigned to the experimental group and 34 to the control group. Allocation was concealed using sequentially numbered, opaque, sealed envelopes prepared by an independent researcher who was not involved in participant recruitment or outcome assessment. Because of the nature of the intervention, participants and the researcher delivering the VR-assisted educational intervention could not be blinded. However, the researcher responsible for administering and collecting the RHDS-SF questionnaires was independent of the intervention delivery and remained blinded to group allocation throughout data collection.

### 2.4. Materials

Questionnaire Form: This questionnaire was developed by the researchers based on the relevant literature [20,21,22] to collect participants’ baseline demographic and clinical characteristics that could potentially influence readiness for hospital discharge. It consists of two separate sections: one covering patient demographics and the second section includes information on the patient’s health status. The first section includes 7 questions covering the patient’s age, place of residence, marital status, economic status, living arrangement, educational level, and access to social support; the second section includes 3 questions regarding previous reasons for hospitalization, chronic disease status, and duration of illness, for a total of 10 questions.

Readiness for Hospital Discharge Scale/Short Form (RHDS-SF): RHDS-SF originally developed by Weiss and Piacentine [7] and adapted into Turkish by Kaya et al. [23], was used to assess patients’ readiness for hospital discharge. The original version demonstrated excellent internal consistency (Cronbach’s α = 0.90), whereas the Turkish version demonstrated acceptable internal consistency (Cronbach’s α = 0.74) and good construct validity (χ^2^/df = 2.6, RMSEA = 0.03, CFI = 1.00, GFI = 0.99, AGFI = 0.99). The RHDS-SF consists of eight items rated on an 11-point Likert scale ranging from 0 (“not at all”) to 10 (“totally”). The overall score is calculated as the mean of the eight items, with higher scores indicating greater readiness for hospital discharge.

### 2.5. Intervention

#### 2.5.1. Development of the Intervention

The VR-assisted discharge education program was systematically developed according to the ASSURE instructional design model, providing a structured framework for the development of a patient-centered educational intervention. The educational content was developed based on a needs assessment supported by current evidence and expert opinion. The needs assessment consisted of informal interviews with urologists, surgical nurses, and patients undergoing TURP to identify the essential educational needs during the postoperative recovery period.

The educational program covered the key components of postoperative self-management, including an introduction to postoperative recovery, catheter care, bleeding management, pain management, nutrition, early mobilization, activity recommendations, medication use, sexuality, warning signs requiring medical attention, and recommendations for home care after discharge. The content was presented using narration, animations, graphics, and visual demonstrations to facilitate understanding and improve knowledge retention. Before implementation, the educational materials were reviewed for scientific accuracy, clinical relevance, and comprehensibility by a urology specialist and a surgical nursing specialist, and the final version was approved for use.

#### 2.5.2. Delivery of the Intervention

Participants allocated to the intervention group received the standardized discharge education individually using an Oculus Quest 2 VR headset. The educational content was delivered audiovisually through over-ear, closed-back headphones, with the sound level standardized at approximately 60–65 dB. The educational program followed a standardized sequence consisting of an introduction to postoperative recovery, catheter care, bleeding management, pain management, nutrition, mobilization, medication use, warning signs requiring medical attention, and recommendations for home care after discharge. All participants received the same educational content under identical conditions. The intervention was administered approximately two hours before surgery in a single session lasting approximately 17.12 min. During the study, the educational video was uploaded to YouTube as an unlisted video and was accessible only via a direct link provided by the research team. The video was made publicly available only after completion of participant recruitment and data collection to promote transparency and facilitate dissemination of the intervention (https://youtu.be/VX5bnhYCqWQ (accessed on 1 June 2026)). Participants were instructed to complete the RHDS-SF based on how prepared they felt for their anticipated discharge following the planned surgery. The baseline assessment was performed before surgery because the educational intervention was delivered preoperatively, when patients were fully conscious, able to concentrate, and more receptive to educational information. To ensure intervention fidelity, all participants in the intervention group received the same standardized educational content using identical VR equipment, audio settings, educational sequence, and implementation procedures delivered by the same researcher.

Patients in the control group received the routine discharge education approximately two hours before surgery, consistent with standard clinical practice. This education was delivered verbally by the ward nurse and included instructions on catheter care, medication use, and seeking medical attention in the event of bleeding. No additional educational intervention or VR-based education was provided. Catheter care and bleeding-related warning signs were addressed in both groups, whereas the VR-assisted program included additional topics related to postoperative self-care and recovery.

### 2.6. Study Procedure

Patients scheduled for TURP in the urology clinic were screened weekly for eligibility according to the study inclusion criteria. Eligible patients were informed about the study, and written informed consent was obtained from those who agreed to participate. Baseline demographic characteristics and pre-test measurements were collected before randomization.

Participants were then randomly assigned to either the intervention or control group by a researcher who was not involved in participant recruitment, intervention delivery, or outcome assessment. Following randomization, patients in the intervention group received the standardized VR-assisted discharge education, whereas those in the control group received routine discharge education according to the hospital’s standard practice.

Post-test data were collected from both groups on the second postoperative day immediately before hospital discharge using the same measurement instruments. The overall study process is illustrated in the CONSORT flow diagram.

### 2.7. Data Analysis

All statistical analyses were performed using IBM SPSS Statistics version 25 (IBM Corp., Armonk, NY, USA). Descriptive statistics were presented as mean ± standard deviation (SD) for continuous variables and as frequency (*n*) and percentage (%) for categorical variables. Internal consistency of the Readiness for Hospital Discharge Scale was evaluated using Cronbach’s alpha coefficients separately for the pre-test and post-test measurements.

The normality of continuous variables was assessed using the Shapiro–Wilk test together with histogram, Q–Q plot, skewness, and kurtosis values. Homogeneity of variances between groups was evaluated using Levene’s test. Baseline demographic and clinical characteristics were compared between the intervention and control groups using the independent samples *t*-test for continuous variables and the chi-square test or Fisher’s exact test for categorical variables, as appropriate.

To evaluate the effect of the intervention on discharge readiness over time, a two-way mixed-design analysis of variance (mixed ANOVA) was performed with group (intervention vs. control) as the between-subjects factor and time (pre-test vs. post-test) as the within-subjects factor. Prior to the mixed-design ANOVA, the assumptions of normality and homogeneity of variances were evaluated. Sphericity was assumed because the within-subject factor consisted of only two measurement occasions. Effect sizes for the mixed-design ANOVA were reported using partial eta squared (ηp^2^). Statistical significance was established at *p* < 0.05.

## 3. Results

A total of 80 patients were assessed for eligibility. Of these, 12 were excluded before randomization: 10 did not meet the predefined eligibility criteria and two declined to participate before randomization. The remaining 68 patients were randomized equally to the intervention (*n* = 34) and control (*n* = 34) groups. All randomized participants completed the study and were included in the analysis, with no losses to follow-up. Baseline demographic and clinical characteristics of the participants are summarized in Table 1. The groups were comparable with respect to marital status, place of residence, education level, perceived income, living arrangement, health insurance status, chronic disease, and receipt of preoperative information (all *p* > 0.05). However, patients in the intervention group were significantly older than those in the control group (63.03 ± 6.59 vs. 58.53 ± 6.83 years, *p* = 0.007). Although participants in the intervention group were significantly older than those in the control group, the intervention group nevertheless demonstrated greater improvements in discharge readiness. Because increasing age is generally associated with lower health literacy and greater educational needs, this baseline imbalance would not be expected to favor the intervention group. Nevertheless, future studies should consider adjustment for baseline covariates.

The RHDS-SF demonstrated acceptable internal consistency at pre-test (Cronbach’s α = 0.705) and good internal consistency at post-test (Cronbach’s α = 0.807), supporting the reliability of the instrument in the present study.

As shown in Table 2, baseline RHDS-SF scores were similar between the intervention and control groups. Following the intervention, readiness for hospital discharge increased in both groups, with a markedly greater increase observed in the intervention group.

The results of the two-way mixed-design ANOVA are presented in Table 3. A significant main effect of time was observed, indicating that readiness for hospital discharge increased significantly from pre-test to post-test across both groups (*F*(1, 66) = 2565.68, *p* < 0.001, ηp^2^ = 0.975). Importantly, a significant time × group interaction indicated that the increase in RHDS-SF scores over time differed significantly between the intervention and control groups (*F*(1, 66) = 107.23, *p* < 0.001, ηp^2^ = 0.619).

No adverse events related to the VR intervention (e.g., cybersickness, dizziness, nausea, or visual discomfort) were observed during the intervention period.

## 4. Discussion

The present randomized controlled trial demonstrated that VR-assisted discharge education was more effective than routine discharge education in improving readiness for hospital discharge among patients undergoing TURP. Although discharge readiness increased in both groups following surgery, the improvement was substantially greater in the intervention group. The significant time × group interaction with a large effect size further suggests that the observed improvement was primarily associated with the intervention rather than the passage of time alone. These findings suggest that integrating immersive educational technologies into discharge planning may enhance patients’ preparedness for self-care during the transition from hospital to home. An important consideration when interpreting these findings is that participants in the intervention group were significantly older at baseline than those in the control group. Despite this baseline difference, the intervention group demonstrated significantly greater improvements in readiness for hospital discharge. Although randomization is expected to minimize systematic differences between groups, chance baseline imbalances may still occur, particularly in studies with relatively small sample sizes. Future studies should consider adjusting for such imbalances using covariate analyses when appropriate. The preoperative administration of the RHDS-SF should be considered when interpreting the findings. In this study, the baseline assessment was conducted before surgery because the discharge education intervention was intentionally delivered during the preoperative period, when patients were fully conscious and better able to engage with educational content. Therefore, baseline scores reflect patients’ anticipated readiness for discharge rather than their actual postoperative readiness, which may have contributed to the relatively low pre-test scores observed. Nevertheless, because both groups were assessed under identical conditions before randomization, this approach provided a consistent baseline for evaluating changes attributable to the intervention.

Several explanations may account for why VR-assisted discharge education produced greater improvements in discharge readiness than routine education. Unlike conventional verbal explanations or written materials, VR provides an immersive and interactive learning environment that promotes experiential learning and encourages patients to actively engage in the educational process. Previous research has shown that active participation enhances attention, information retention, motivation, and comprehension of complex health information, thereby facilitating more effective learning experiences [17,18]. In the present study, the visual demonstration of postoperative self-care practices—including catheter management, early mobilization, and recognition of warning signs—may have increased patients’ confidence in performing these activities after discharge. Therefore, the observed benefits are likely attributable not only to the use of VR technology itself but also to its ability to deliver educational content in a more engaging, understandable, and memorable manner. Furthermore, providing the educational intervention before surgery may have allowed patients to process and retain postoperative self-care information before the immediate postoperative period, when pain, fatigue, and residual anesthetic effects could interfere with learning. This timing may have contributed to better cognitive processing of educational content and greater preparedness for postoperative self-care [14].

The findings of the present study are consistent with previous evidence demonstrating that structured educational and supportive interventions improve postoperative recovery and self-care among patients undergoing TURP. Recent studies have shown that nurse-led educational interventions, continuing care programs, and structured postoperative support can improve self-care ability, functional recovery, quality of life, and reduce postoperative complications following TURP. In particular, educational interventions incorporating individualized self-management training and follow-up have been associated with better symptom control, improved functional outcomes, and greater patient confidence in managing recovery after discharge. These findings support the importance of providing comprehensive patient education as an integral component of postoperative care for patients undergoing TURP [4,24,25].

However, unlike previous studies that primarily evaluated quality of life, symptom management, functional recovery, or self-care behaviors, the present study focused on readiness for hospital discharge as the primary outcome. Readiness for hospital discharge has increasingly been recognized as an important indicator of successful transitional care because it reflects patients’ confidence and preparedness to continue recovery safely after leaving the hospital [9,10]. By demonstrating that VR-assisted discharge education significantly improves discharge readiness, the present study extends the current evidence and provides additional support for incorporating immersive educational technologies into perioperative patient education.

The positive outcomes observed in this study may be attributable not only to the use of VR technology but also to the systematic development of educational intervention. The discharge education program was designed according to a structured instructional framework based on patients’ learning needs and current evidence. This approach enabled the educational content to address practical postoperative challenges, including catheter management, medication use, early mobilization, pain management, nutrition, and recognition of potential complications. Educational interventions developed through systematic instructional design have been shown to improve learner engagement, facilitate knowledge acquisition, and enhance the effectiveness of technology-supported education [26]. Likewise, recent studies have emphasized that the effectiveness of digital patient education depends not only on the technology itself but also on the quality, relevance, and organization of the educational content [15]. Therefore, the favorable outcomes observed in the present study are likely related to both the immersive characteristics of VR and the structured design of the educational program.

The findings of this study also have important implications for clinical practice. Advances in surgical techniques and the widespread implementation of enhanced recovery after surgery (ERAS) pathways have shortened hospital stays, requiring patients to assume greater responsibility for postoperative self-care soon after discharge [17,19,27]. Consequently, healthcare professionals must provide comprehensive discharge education within increasingly limited timeframes. In this context, VR-assisted education offers an opportunity to deliver standardized, visually rich, and immersive educational experiences that may facilitate understanding, improve information retention, and strengthen patients’ confidence in managing their recovery at home. Furthermore, supporting patients during the transition from hospital to home has been recognized as a key component of high-quality perioperative care and is closely associated with safer recovery and improved patient outcomes [6]. Therefore, rather than replacing conventional discharge education, VR should be considered a complementary educational strategy that may strengthen patient-centered discharge planning and postoperative care [15]. However, implementation in routine care also requires consideration of the initial costs of VR equipment, software and educational content development, as well as ongoing maintenance and updating requirements. As cost-effectiveness was not evaluated in the present study, further studies should examine whether the educational benefits of VR justify these costs and support its sustainable integration into routine discharge care.

### Strengths and Limitations

This study has several methodological strengths. First, the randomized controlled design strengthened the internal validity of the findings by reducing the risk of selection bias. Second, the intervention was developed through a structured, needs-based instructional design process and delivered using standardized educational materials, identical VR equipment, and a consistent implementation protocol, thereby ensuring intervention fidelity. Third, readiness for hospital discharge was assessed using a validated measurement instrument that demonstrated acceptable to good internal consistency across the two measurement points. Finally, to our knowledge, this is among the first randomized controlled trials to evaluate the effect of VR-assisted discharge education on readiness for hospital discharge in patients undergoing TURP, thereby providing novel evidence for the integration of immersive educational technologies into perioperative patient education.

Several limitations should be considered when interpreting the findings of this study. First, this was a single-center study, which may limit the generalizability of the findings to different healthcare settings and patient populations. In addition, the acceptability and effectiveness of VR-assisted education may vary according to patients’ educational and sociocultural backgrounds and their familiarity with digital technologies. This may be particularly relevant for older adults with limited technology literacy, and should be considered when adapting VR-based discharge education to different patient populations. Second, readiness for hospital discharge was assessed only immediately before discharge, and patients were not followed after hospital discharge. Therefore, 30-day post-discharge outcomes, including hospital readmissions, emergency department visits, and postoperative complications such as catheter-related problems or infections, were not systematically tracked. Consequently, although VR-assisted education improved patients’ perceived readiness for discharge, the present study cannot determine whether this improvement translated into fewer post-discharge complications, healthcare utilization, or other longer-term clinical benefits. Third, because the nature of the intervention did not permit participant blinding, the possibility of performance bias cannot be completely excluded. In addition, participants’ awareness of receiving a novel VR-based educational intervention and of participating in a research study may have introduced expectation or novelty bias, whereby the intervention group perceived themselves as better prepared because of the innovative nature of the intervention or the increased attention received. Given that readiness for hospital discharge was assessed using a self-reported measure, these perceptions may have influenced participants’ responses in addition to their actual preparedness. Therefore, although the observed effect sizes were substantial, they should be interpreted with consideration of this potential source of bias. In addition, because the VR-assisted program provided broader and more standardized educational content than routine discharge education, the observed improvement cannot be attributed solely to the VR delivery modality. Differences in the scope, content, and standardization of the educational intervention may also have contributed to the between-group differences. Future multicenter randomized controlled trials with longer follow-up periods are warranted to determine whether improvements in discharge readiness are associated with sustained clinical benefits and better post-discharge outcomes, as suggested in previous transition-of-care research [9,10].

## 5. Conclusions

The structured VR-assisted discharge education intervention was more effective than routine discharge education in improving patients’ perceived readiness for hospital discharge under the conditions of this study. This randomized controlled trial demonstrated that VR-assisted discharge education significantly improved readiness for hospital discharge in patients undergoing TURP compared with routine discharge education. These findings suggest that a structured VR-assisted discharge education intervention may enhance patients’ perceived readiness for hospital discharge compared with routine discharge education.

As hospital stays continue to shorten and patients are expected to assume greater responsibility for their recovery after discharge, innovative and patient-centered educational approaches are becoming increasingly important. VR-assisted discharge education may serve as a practical adjunct to conventional discharge teaching in perioperative nursing care. Future multicenter randomized controlled trials are warranted to determine whether improvements in discharge readiness translate into sustained improvements in postoperative self-care, quality of life, complications, and hospital readmissions.

## Figures and Tables

**Table 1:** Baseline demographic and clinical characteristics of participants by study group.

Characteristics	Control Group (*n* = 34)	Intervention Group (*n* = 34)	*p* Value
**Age (years), mean ± SD**	58.53 ± 6.83	63.03 ± 6.59	0.007
**Marital status, *n* (%)**			0.673 *
Married	32 (94.1)	30 (88.2)	
Single	2 (5.9)	4 (11.8)	
**Place of residence, *n* (%)**			0.193
Village	16 (47.1)	18 (52.9)	
District	11 (32.4)	5 (14.7)	
City	7 (20.6)	11 (32.4)	
**Education level, *n* (%)**			0.104
Literate	16 (47.1)	15 (44.1)	
Primary school	6 (17.6)	14 (41.2)	
Secondary school	9 (26.5)	4 (11.8)	
High school	3 (8.8)	1 (2.9)	
**Perceived income level, *n* (%)**			0.576
Poor	16 (47.1)	12 (35.3)	
Moderate	14 (41.2)	16 (47.1)	
Good	4 (11.8)	6 (17.6)	
**Living arrangement, *n* (%)**			0.114 *
Alone	0 (0.0)	4 (11.8)	
With spouse	34 (100.0)	30 (88.2)	
**Health insurance status, *n* (%)**			0.186 *
No	3 (8.8)	8 (23.5)	
Yes	31 (91.2)	26 (76.5)	
**Chronic disease, *n* (%)**			0.560 *
No	6 (17.6)	9 (26.5)	
Yes	28 (82.4)	25 (73.5)	
**Received preoperative information, *n* (%)**			0.689 *
No	31 (91.2)	28 (82.4)	
Yes	3 (8.8)	6 (17.6)	

* Indicates Fisher’s exact test. *p* values were calculated using the independent samples *t*-test for age and the Pearson chi-square test for categorical variables.

**Table 2:** Changes in Readiness for Hospital Discharge Scale scores before and after the intervention.

RHDS-SF Measurement	Control Group (*n* = 34) Mean ± SD	Intervention Group (*n* = 34) Mean ± SD
**Pre-test**	3.44 ± 0.52	3.36 ± 0.48
**Post-test**	6.48 ± 0.46	7.95 ± 0.41

**Note:** RHDS-SF = Readiness for Hospital Discharge Scale- Short Form; SD = standard deviation. Higher scores indicate greater readiness for hospital discharge.

**Table 3:** Mixed-design ANOVA results for readiness for hospital discharge.

Effect	F	*p*	Partial η^2^
Time	2565.68	<0.001	0.975
Time × Group	107.23	<0.001	0.619

**Note:** Partial η^2^ values of 0.01, 0.06, and 0.14 represent small, medium, and large effect sizes, respectively.

## Data Availability

The datasets generated and/or analyzed during the current study are available from the corresponding author upon reasonable request.

## Abbreviations

ANOVA	Analysis of Variance
CONSORT	Consolidated Standards of Reporting Trials
ERAS	Enhanced Recovery After Surgery
RHDS-SF	Readiness for Hospital Discharge Scale–Short Form
SD	Standard Deviation
TURP	Transurethral Resection of the Prostate
VR	Virtual Reality
